# An anatomically-based masking protocol for the assessment of in-shoe plantar pressure measurement of the forefoot

**DOI:** 10.1186/s13047-018-0271-4

**Published:** 2018-06-15

**Authors:** Saeed Forghany, Daniel R. Bonanno, Hylton B. Menz, Karl B. Landorf

**Affiliations:** 1 Musculoskeletal Research Centre, School of Rehabilitation Sciences, Isfahan University of Medical Sciences, Isfahan, Iran; 2 Centre for Health Sciences Research, School Health Sciences, University of Salford, Salford, UK; 3 Discipline of Podiatry, School of Allied Health, La Trobe University, Melbourne, VIC 3086 Australia; 4 La Trobe Sport and Exercise Medicine Research Centre, School of Allied Health, La Trobe University, Melbourne, VIC 3086 Australia

**Keywords:** Forefoot, Gait, Kinetics, Orthotic devices

## Abstract

**Background:**

The area beneath the metatarsal heads is a common location of foot pain, which is often associated with high plantar pressures. Current plantar pressure assessment protocols focus mainly on the gross area of the forefoot with minimal attention paid to specific areas such as the metatarsal heads. The aim of this study was to develop and assess a new anatomically-based masking protocol that is clinically relevant to measure forefoot plantar pressure during shod conditions based on the anatomical positions of the metatarsal heads.

**Methods:**

Initially, we developed a masking protocol to measure forefoot plantar pressure during shod conditions based on the anatomical positions of the metatarsal heads. This new masking protocol divided the forefoot into three sub-areas (proximal, beneath, and distal to the metatarsal heads) as determined by the position of each metatarsal head. Following development of the new masking protocol, we compared the new protocol against a traditional protocol, which defines the forefoot as between 51 and 81% of the foot length. To compare the two masking protocols, we tested two experimental conditions: (i) a control condition (i.e. no metatarsal pad), and (ii) a metatarsal pad condition. We then compared plantar pressure differences between the two experimental conditions for the two masking protocols. Participants for this component of the study included 36 community dwelling older adults (mean age 75.6 years ±5.4) with a history of forefoot pain. Forefoot plantar pressure data were measured while walking using the pedar^®^-X in-shoe system. Peak pressure, maximum force and contact area at the time of peak pressure were determined and results were compared between the two masking protocols.

**Results:**

The traditional masking protocol showed that the metatarsal pad significantly decreased peak pressure and increased contact area in the forefoot area (i.e. within the entire mask area), but maximum force was not significantly different between the two conditions. In contrast, the newly developed anatomically-based masking protocol indicated that the metatarsal pad decreased peak plantar pressures distal to and beneath the metatarsal heads by increasing force and contact area proximal to the metatarsal heads.

**Conclusions:**

An anatomically-based masking protocol that is clinically relevant was developed to assess forefoot plantar pressure during shod conditions based on the anatomical positions of metatarsal heads. We propose that the new forefoot masking protocol will provide greater interpretability of forefoot plantar pressure data, which will aid clinicians and researchers for diagnostic, prognostic and therapeutic purposes.

## Background

The area beneath the metatarsal heads is a common location of forefoot pain, calluses and neuropathic foot ulcers, and such conditions are often associated with high plantar pressures [1–4]. Accordingly, plantar pressure measurement is frequently recommended as a tool to guide diagnosis, treatment and research investigation for such conditions [5]. There is a relatively large volume of literature that relates to plantar pressure characteristics in the areas of the metatarsal heads, which also includes plantar pressure changes due to treatment of these conditions [2, 6–10]. However, variability in the selection of areas under the metatarsal heads to be investigated on plantar pressure images − referred to as masking − makes explaining plantar pressure changes difficult.

In most plantar pressure studies, the forefoot is selected as a percentage of the plantar pressure insole’s length [7, 8, 11–15]. This technique has been claimed to have been determined from skeletal anatomy, but evidence for these percentage measures is sparse. These percentage measures could also be misleading in the presence of foot deformities and during shod conditions, as the pressure measuring insoles are often a different length to the actual foot length being investigated (i.e. they are matched to shoe length rather than foot length). As such, the data from such studies could be ambiguous as it is not clear exactly which part of the foot the pressure results reflect, particularly in relation to the metatarsal heads.

An alternative method of anatomical masking of plantar pressure images involves the synchronisation of a pressure platform with a 3-dimensional motion capture system [16, 17]. Reflective markers are placed on relevant anatomical landmarks on the foot (e.g. the metatarsal heads) and the position of each marker is projected vertically onto the plantar pressure images. Sub-divisions of the foot are then determined based on the position of the markers. However, this method is unlikely to be feasible in clinical scenarios due to its complexity and the high cost of setting up such a system.

Clearly, further research is needed to develop and test appropriate techniques for masking the forefoot to ensure that the most useable information is gained from plantar pressure assessment. These techniques also need to be relatively easy to perform and not be reliant on complex and expensive laboratory techniques. The aim of this study was to develop and assess a new anatomically-based forefoot masking protocol (based on the anatomical positions of the metatarsal heads) that is clinically relevant to measure plantar pressure during shod conditions. To achieve this aim, the effect of a metatarsal pad on forefoot plantar pressure was investigated to evaluate the new masking protocol.

## Methods

The methods for this study are broken into two distinct sections. Initially, the development of the new masking protocol is outlined. Following this, the assessment of the new protocol (reliability and comparison with the traditional masking protocol) is detailed.

### Development of the new masking protocol

For the purpose of developing the new anatomically-based masking protocol, the following method was used. Firstly, each participant’s shoe size was determined and they were fitted with a pair of standard extra-depth shoes (Gadean^®^, Perth, Australia). Secondly, following determination of the correct shoe size, a pre-made thin (< 1.0 mm), flexible cardboard insole (also referred to as a template) was fitted to the shoe; multiple templates were pre-made for different shoe sizes. Minor adjustments were made with scissors to ensure that the dimensions of the cardboard insole matched the shoe.

The thin cardboard insole was made for each participant for the purpose of determining the location of the metatarsal heads relative to the insole of the shoe. Accordingly, after fitting the cardboard insole, the metatarsal heads were then palpated and marked with an ink pen on the plantar skin surface, similar to the technique validated by Spooner and colleagues [18]. The participant then placed their feet inside the shoes (i.e. with the cardboard insole inside) and with the laces fastened, the participant was asked to stand to allow the ink to transfer from the foot to the cardboard insole. This process ensured that the position of each metatarsal head (i.e. the inked mark) was transferred to the cardboard insole. The participant was then asked to remove the shoes so that the outline of the metatarsal parabola could be marked on the cardboard insole (from the position of the metatarsal heads outlined by the ink marks).

Once the cardboard insole was marked, the positions of each metatarsal head relative to the insole length was determined. To achieve this, we developed the following standardised protocol.

### Step (i)

**Fig. 1:**
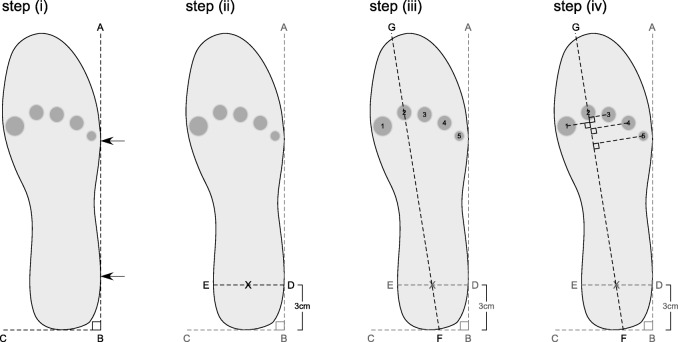
Protocol to determine the positions of each metatarsal head relative to the cardboard insole length

To begin, a *lateral reference line* was determined so each cardboard insole could be aligned in a standardised position (Fig. 1). The lateral reference line was created by drawing a line between two points: (i) a point representing the most lateral aspect of the heel, and (ii) a point representing the most lateral aspect of the forefoot of the insole. This line is represented by line AB in Fig. 1, step (i).

### Step (ii)

Next, a *medial to lateral heel line* was added to the cardboard insole. With the insole positioned in the standardised position outlined above, the medial to lateral heel line was drawn perpendicular to the lateral reference line at a point 3 cms distal to the most proximal aspect of the heel. This line is represented by line DE in Fig. 1, step (ii). The middle of the heel was then defined as the mid-point along the medial to lateral heel line.

### Step (iii)

Next, a line representing a *longitudinal axis line* was added to the cardboard insole. This line was established by passing a straight line along the length of the insole that passed through the mid-point of the heel line and orientated distally through the second metatarsal head. This line is represented by line FG in Fig. 1, step (iii). The length of the insole was then measured along the longitudinal axis from the most proximal point (point F) to the most distal point (Point G) of the template.

### Step (iv)

Finally, the *relative position of each metatarsal head* was projected onto the longitudinal axis line and their position relative to the cardboard insole length (i.e. relative length) was quantified. To achieve this, the middle of each metatarsal head was marked and a line was drawn from this point so that it intersected with the longitudinal axis at a right angle. This step is represented in Fig. 1, step (iv).

### Participants

Thirty six community-dwelling older people aged 65 or older with the history of forefoot pain were recruited. Participants were recruited and tested between March and November 2013. Ethics approval was obtained from the La Trobe University Faculty Human Ethics Committee – application FHEC12/207. All participants signed informed consent prior to recruitment into the study.

### Sample size determination

The sample size was determined prior to conducting the study (i.e. a priori) using an appropriate formula [19]. A sample size of 36 provides an 80% probability of detecting a clinically meaningful difference between interventions of 60 kilopascals (kPa) in peak plantar pressure. The standard deviation used to determine this sample size was taken from a similar studies that measured plantar pressures in older people [7, 8, 20, 21] and was set at 90 kPa. The alpha level was set at 0.05.

### Assessment of the new masking protocol

**Fig. 2:**
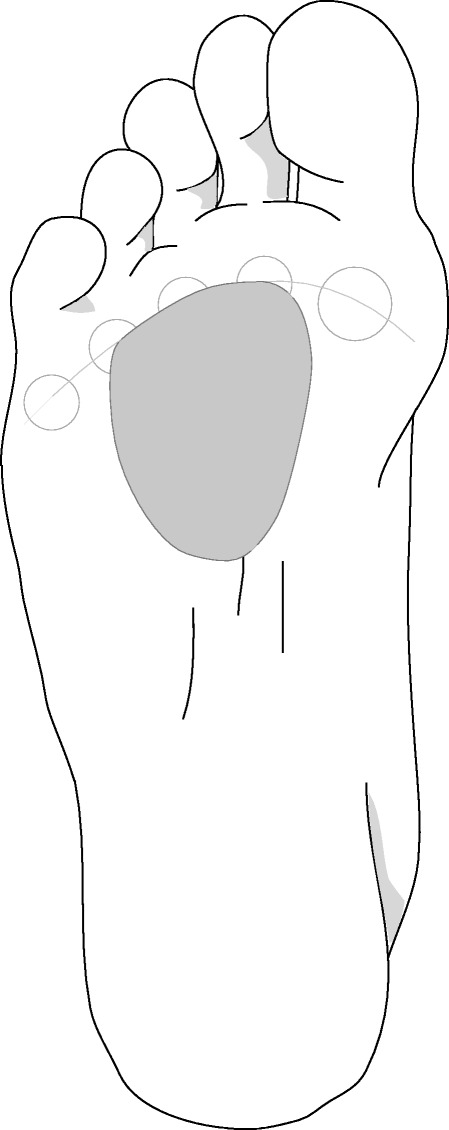
Illustration of the metatarsal pad position relative to the metatarsal head parabola

To assess the new masking protocol, a commercially available prefabricated metatarsal pad constructed from PPT^®^ – a proprietary medical grade soft-tissue supplement – was used (Langer Biomechanics, New York, USA). This pad is teardrop shaped and is 6 mm at the highest point. It is a commonly used pad to alleviate plantar forefoot pain and the supplier of the pads for this study indicated that it is the most commonly ordered metatarsal pad from their company by podiatrists in Australia (personal communication, Mark Dannals, Briggate Medical Company). The pad was dispensed in one of two different sizes (small or medium) depending on foot size. When the metatarsal pad was tested, it was adhered to the cardboard insole using double-sided adhesive tape to prevent the pad from moving during testing. The cardboard insole was positioned between the plantar surface of the foot and the inside of the sole of the shoe. There were two conditions tested in this study:

(i) control (no pad) condition;
(ii) metatarsal pad positioned in line with the metatarsal heads (Fig. 2).

The order of testing was randomised to avoid ordering effects. In addition, we attempted to blind participants as to the types of interventions used (this was part of a larger study where multiple pads were evaluated), so participants were not informed of the study purpose, other than that we were evaluating how forefoot pads change pressure under the foot. Accordingly, participants were not advised of the study hypotheses.

Plantar pressures beneath the foot were measured using the pedar^®^-X in-shoe plantar pressure system (Novel GmbH, Munich, Germany). The pedar^®^-X comprises of 99 capacitive sensors arranged in a grid and embedded within a thin flexible insole. The pedar^®^-X insoles were calibrated using the trublu^®^ calibration device (Novel GmbH, Munich, Germany) [22] prior to data collection. The sampling frequency of the system was 50 Hz. The pedar^®^-X is widely used in foot plantar pressure research [6, 21, 23–25] and has been demonstrated to be a valid and reliable in-shoe pressure measurement system [26–29]. It has high test-retest reliability with coefficients of repeatability for metatarsal head measurements of between 1.2 to 7.7% [28], and coefficients of variation for metatarsal head measurements of between 3.4 and 24.1% [29]. This equipment has also previously been used in similar projects with older people [7, 8, 20, 21].

The appropriately sized pedar^®^-X insole was inserted into the shoe on top of the cardboard insole, and then the participant put the shoes on again, so the pedar^®^-X insole was positioned between the participant’s foot and the cardboard insole with the metatarsal pad. The participant was then instructed to walk at their normal comfortable speed while being timed. To minimise the confounding effect of different walking speeds on the pressure data, the trial was repeated if the walking speed differed by more than 5% of the original walking speed. Four walking trials along an 8 m walkway were recorded for each test condition, with the middle 4 steps for each trial included in the analysis (to exclude acceleration and deceleration steps). The 16 steps (4 trials × 4 steps) were then averaged for each condition.

**Fig. 3:**
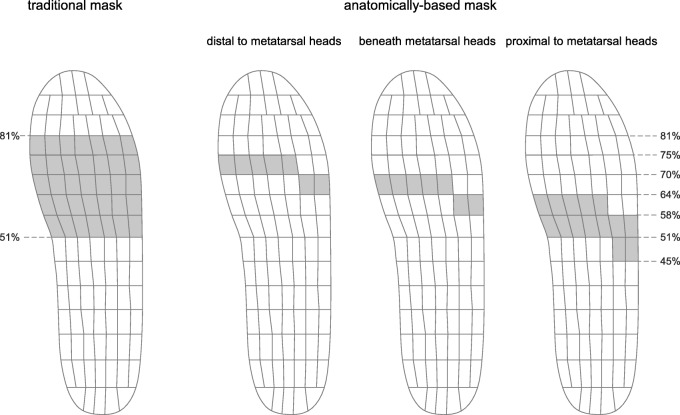
The traditional masking protocol and the anatomically-based masking protocol (Note: for the anatomically-based protocol, three mask sub-areas of forefoot were selected according to the positions of metatarsal heads: (i) distal to the metatarsal heads, (ii) beneath the metatarsal heads, and (iii) proximal to the metatarsal heads)

The forefoot plantar pressure data were then assessed with a traditional masking protocol that uses the percentage of foot length (between 51 and 81% of foot length) [22]. This was then compared with our new masking protocol (Fig. 3). To determine the new masking protocol, the following process was followed. Once the relative position of each metatarsal head was calculated from the cardboard insole (i.e. % relative to the insole length), this information was used to determine which sensors on the pedar^®^-X insole were to be analysed for the ‘beneath the metatarsal heads’ analysis. Once this was determined, the sensors one row distal to this were chosen for the ‘distal to the metatarsal heads’ analysis and the sensors proximal to this were chosen for the ‘proximal to the metatarsal heads’ analysis. Two rows of sensors were chosen for the ‘proximal to the metatarsal heads’ analysis to allow greater sensor area to detect changes in pressure variables as the metatarsal pad being tested was moved proximally where the arch of the foot was.

The primary outcome measure of interest was peak pressure under the forefoot. The secondary outcomes included maximum force and contact area at the time of peak pressure, which we used to help explain the peak plantar pressure findings. Outcomes were determined using both masking protocols and then the results from each protocol were compared.

### Data analysis

To analyse reliability of the measurements from the cardboard insoles the following was done. Intra-rater reliability was analysed by examining repeated measurements of cardboard insole length and metatarsal head length relative to cardboard insole length. Measurements were taken over two different days on the same cardboard insoles by the same two raters (DRB and KBL). Inter-rater reliability was analysed by measurement of cardboard insole length and positions of the metatarsal heads by three different raters (SF, DRB and KBL) who took the measurements independent of each other. Intra-class correlation coefficients (ICCs) were used to determine the reliability coefficients using an ICC (3,1) model [30].

To analyse the plantar pressure data, we firstly checked that the plantar pressure data were normally distributed by assessing skewness, kurtosis, and the Shapiro–Wilk test (*p* > 0.05). To compare the two masking protocols, paired *t*-tests were used to evaluate for differences (*p* < 0.05) between the two conditions (i.e. no pad control condition versus metatarsal pad condition).

IBM SPSS Statistics version 16.0 (IBM Corporation, Armonk, NY, USA) was used for all analyses, except for percentage change and effect size calculations, which were carried out with Microsoft Excel (Microsoft Office 2013) using the formula proposed by Thalheimer and Cook [31]. Effect size magnitudes were categorised according to Hopkins [32].

## Results

### Participant characteristics

The mean age of participants was 75.6 ± 5.4 years (range 65.1 to 88.6 years). There were 31 females and 5 males. The mean BMI for participants was 28.3 ± 4.1 kg/m^2^.

### Anatomically-based masking protocol

An anatomically-based masking protocol was developed by dividing the forefoot into three sub-areas as determined by the position of the metatarsal heads. Metatarsal heads were located between 59.7 ± 1.8% and 69.9 ± 1.8% of cardboard insole length. Reliability analysis indicated that the protocol had excellent intra- and inter-rater reliability. Table 1 presents the ICC values for the intra- and inter-rater reliability analysis for cardboard insole length and metatarsal heads positions.

**Table 1:** ICC values of between-day and between-tester for cardboard insole length and metatarsal head positions

	R1D1 vs R1D2	R2D1 vs R2D2	R1D1 vs R2D1	R1D2 vs R2D2	R1D1 vs R2D1 vs R3D1
Insole length	0.989	0.998	0.987	0993	0.995
Metatarsal head 1	0.964	0.996	0.966	0.987	0.982
Metatarsal head 2	0.990	0.998	0.985	0.991	0.989
Metatarsal head 3	0.988	0.996	0.988	0.988	0.993
Metatarsal head 4	0.981	0.988	0.983	0.973	0.980
Metatarsal head 5	0.983	0.991	0.984	0.979	0.967

*R*1 Rater 1, *R*2 Rater 2, *R*3 Rater 3, *D*1 Day 1, *D*2 Day 2

Table 2 presents the mean position of each metatarsal head as a percentage of the overall cardboard insole length.

**Table 2:** Position of each metatarsal head as the percentage of cardboard insole length (values are means ± SDs)

	Metatarsal head 1	Metatarsal head 2	Metatarsal head 3	Metatarsal head 4	Metatarsal head 5
Position (% of cardboard insole length)	69.3 ± 1.9	69.9 ± 1.8	67.7 ± 1.7	64.0 ± 1.6	59.7 ± 1.8

Three mask sub-areas of forefoot were selected according to the positions of metatarsal heads: (i) distal to the metatarsal heads, (ii) beneath the metatarsal heads, and (iii) proximal to the metatarsal heads. The traditional masking protocol and the anatomically-based masking protocol, including the three mask sub-areas, are illustrated in Fig. 3.

### Traditional masking protocol versus anatomically-based masking protocol

In relation to peak pressure (Table 3), analysing data using the traditional masking protocol showed that the metatarsal pad (compared to the control condition) significantly reduced peak pressure in the forefoot (− 14.3%, *p* < 0.001, effect size medium 0.55). Analysing data using the anatomically-based masking protocol showed that the metatarsal pad (compared to the control condition) significantly reduced peak pressure in the forefoot mask sub-areas *distal* to the metatarsal heads (− 17.0% change, *p* < 0.001, effect size medium 0.55) and *beneath* the metatarsal heads 3 (− 17.3% change, *p* = 0.004, effect size medium 0.53). However, peak pressure did not change significantly in the forefoot mask sub-area *proximal* to the metatarsal heads (*p* = 0.333).

**Table 3:** Peak pressure (kPa) using two different masking protocols and during two insole conditions (values are means ± SDs, percentage changes and effect sizes)

		Insole condition		% change (95% CI)	Effect size
		Control	Pad in-line		
Traditional mask		389.4 ± 113.6	333.6 ± 91.2	−14.3% (−20.0 to −8.7%)	0.55*
Anatomically-based mask	Distal	351.7 ± 127.5	292.0 ± 99.0	−17.0% (−24.0 to −9.9%)	0.53*
	Beneath	213.8 ± 90.3	176.8 ± 64.3	−17.3% (− 28.5 to −6.1%)	0.48*
	Proximal	110.9 ± 52.4	104.7 ± 27.7	−5.6% (− 17.3 to 6.0%)	0.15

Note: Control insole condition indicates no metatarsal pad and pad-in-line condition indicates metatarsal pad positioned in-line with the metatarsal head parabola. *Significantly different peak pressure for the pad in-line condition compared with the control condition, *p* < 0.05

In relation to maximum force at the time of peak pressure (Table 4), analysing data using the traditional masking protocol showed that the metatarsal pad (compared to the control condition) did not change maximum force significantly in the forefoot (*p* = 0.115). Analysing data using the anatomically-based protocol showed that the metatarsal pad (compared to the control condition) significantly reduced maximum force at the time of peak pressure in the forefoot mask sub-areas *distal* to the metatarsal heads (− 9.9% change, *p* < 0.001, effect size medium 0.51) and *beneath* the metatarsal heads (− 16.6% change, *p* = 0.001, effect size medium 0.55). There was also a significant increase of maximum force at the time of peak pressure in the forefoot mask sub-area *proximal* to the metatarsal heads (+ 24.5% change, *p* = 0.048, effect size small 0.38).

**Table 4:** Maximum force at the time of peak pressure (Newtons) using two different masking protocols and during two insole conditions (values are means ± SDs, percentage changes and effect sizes)

		Insole condition		% change (95% CI)	Effect size
		Control	Pad in-line		
Traditional mask		518.6 ± 102.1	504.4 ± 91.9	−2.7% (−6.2 to 0.7)	0.15
Anatomically-based mask	Distal	207.3 ± 43.8	186.7 ± 37.9	−9.9% (−13.7 to − 6.1)	0.51*
	Beneath	134.4 ± 50.2	112.0 ± 30.3	−16.6% (− 26.0 to − 7.3)	0.55*
	Proximal	82.0 ± 61.1	102.2 ± 46.2	24.5% (0.2 to 48.9)	0.38*

Note: Control insole condition indicates no metatarsal pad and pad-in-line condition indicates metatarsal pad positioned in-line with the metatarsal head parabola. *Significantly different peak pressure for the pad in-line condition compared with the control condition, *p* < 0.05

In relation to contact area at the time of peak pressure (Table 5), analysing data using the traditional masking protocol showed that the metatarsal pad (compared to the control condition) significantly increased contact area in the forefoot (+ 8.1% change, *p* < 0.001, effect size medium 0.61). Analysing data using the anatomically-based protocol showed that the metatarsal pad (compared to the control condition) did not change the contact area significantly in the forefoot mask sub-area *distal* to the metatarsal heads (*p* = 0.142), but significantly reduced contact area in the forefoot mask sub-area *beneath* the metatarsal heads (− 2.3%, *p* = 0.033, effect size small 0.31) and significantly increased contact area in the forefoot mask sub-area *proximal* to the metatarsal heads (+ 21.8%, *p* < 0.001, effect size medium 0.62).

**Table 5:** Contact area (cm^2^) at the time of peak pressure using two different masking protocols and during two insole conditions (values are means ± SDs, percentage changes and effect sizes)

		Insole condition		% change (95% CI)	Effect size
		Control	Pad in-line		
Traditional mask		40.3 ± 5.9	43.6 ± 5.0	8.1% (4.7 to 11.5)	0.61*
Anatomically-based mask	Distal	10.7 ± 0.9	10.8 ± 0.7	1.2% (−0.4 to 2.9)	0.13
	Beneath	10.1 ± 0.6	9.9 ± 0.7	−2.3% (−4.4 to −0.2)	0.31*
	Proximal	14.7 ± 5.7	17.9 ± 4.7	21.8% (10.6 to 33.0)	0.62*

Note: Control insole condition indicates no metatarsal pad and pad-in-line condition indicates metatarsal pad positioned in-line with the metatarsal head parabola. *Significantly different peak pressure for the pad in-line condition compared with the control condition, *p* < 0.05

## Discussion

To address the methodological shortcomings of existing plantar pressure masking protocols, a new masking protocol was developed to be able to better interpret forefoot plantar pressure during shod conditions based on the anatomical positions of the metatarsal heads. This method allows plantar pressure parameters to be determined proximal, beneath and distal to the metatarsal heads. Our new protocol is tailored to the anatomy of a person’s foot rather than the size of the plantar pressure insole used to assess that person. Importantly, we found that our new masking protocol has excellent intra- and inter-rater reliability.

To highlight the clinical relevance of this method and to help validate our protocol, we focused on the example of forefoot pain in our study. Forefoot pads are often used as they have been shown to reduce plantar pressures under the metatarsal heads, which can alleviate pain under the forefoot. However, the precise mechanism as to how forefoot pads achieve this has remained largely unexplained in the current literature. Research thus far has not been able to accurately map the changes in force and contact area that lead to these plantar pressure changes. Doing so would help understand how forefoot pads work, which may lead to the development of more effective pads.

Our findings also highlight critical deficiencies in the results of plantar pressure measures obtained from traditional masking protocols. When analysing plantar pressure data using the traditional masking protocol, we were not able to determine the effects of the metatarsal pad from the perspective of force and contact area changes. However, analysing pressure data using our new anatomically-based masking showed how a metatarsal pad redistributes plantar pressure – via force and contact area changes – in the forefoot area, particularly in relation to the metatarsal heads. Specifically, the metatarsal pad decreases peak plantar pressures distal to and beneath the metatarsal heads by increasing force and contact area proximal to the metatarsal heads. This highlights the importance of appropriate masking to explain plantar pressure changes in the forefoot and has the potential for more objective evaluation of forefoot problems and interventions for these problems. Our anatomically-based masking will enable researchers and clinicians to target the appropriate area of the plantar surface of the forefoot during research, rehabilitation, and the prescription, design and evaluation of footwear and foot orthoses.

Furthermore, our individualised plantar pressure masking protocol is in-line with individualised orthotic management [33]. Ideally, a forefoot pad would be positioned and assessed using plantar pressure data that is specific to an individual. Doing so should more effectively relieve pain and protect vulnerable tissues. Indeed, the *Guidance on footwear and offloading 2015* document published by the International Working Group on the Diabetic Foot recommends such an approach [34]. Although we did not evaluate patient-reported outcomes, it is plausible that taking such an individualised approach may also improve function, which could lead to benefits to overall foot health status. Such a hypothesis needs to be evaluated in randomised trials.

Our new anatomically-based masking protocol provides a robust technique to measure the positions of the metatarsal heads. Even though we only measured the positions of the metatarsal heads in this study, the proposed method could be extended to the determination of other anatomical landmarks of the plantar surface of the foot (e.g. the styloid process or the medial calcaneal tubercle).

One of most important advantages of plantar pressure measurement devices compared to other gait analysis tools is that they are pre-calibrated, easy to set-up, and portable. Some studies have proposed synchronisation of a pressure platform with a 3-dimensional motion capture system for the purpose of masking [16, 17]. However, this method is cumbersome, especially in clinical scenarios, due to the high costs of set-up and complexity. Further, when using this method the sub-divisions of the foot are determined based on the position of skin markers, but this introduces limitations in marker set-up.

There are four limitations to our new forefoot masking protocol. Firstly, the accuracy of our masking protocol depends on the spatial resolution of the plantar pressure insoles, particularly given that we are using relatively small masking areas [35]. Insoles with large sensors may not offer sufficient spatial resolution, which may render the process inaccurate. This is an inherent limitation of currently available plantar pressure measuring insole technology and can only be solved by manufacturers of such systems improving the spatial resolution by increasing the number of sensors per insole. Indeed, platform-based plantar pressure systems, such as the emed^®^, do just that – they have better spatial resolution because they have more sensors per unit area – but they cannot be used for in-shoe insole evaluations. Secondly, the proposed masking protocol depends on the anatomical knowledge of the clinician (i.e. the initial palpation and marking of the metatarsal heads) and the accuracy with which each anatomical landmark is identified. Further accuracy studies investigating multiple clinicians are suggested to validate our new forefoot masking method using a gold-standard such as weight bearing x-rays. Thirdly, we used standardised extra depth footwear for testing participants to control for variation in footwear and to ensure the process of making and fitting the thin cardboard insoles was as efficient as possible. However, this may have an effect on the generalisability of our findings as outside of our study environment, patients would use their own shoes. Finally, our study included a relatively small sample of participants. Studies on larger samples of people with normal foot structure/alignment and those with orthopaedic deformity are suggested to provide more extensive validation of our new protocol. This will enable us to target specific areas of the plantar surface of the foot appropriately.

## Conclusion

An easy to use and anatomically-based masking protocol that is clinically relevant was developed to assess forefoot plantar pressure during shod conditions based on the anatomical positions of metatarsal heads. The new protocol was found to have excellent intra- and inter-rater reliability. We propose that the new forefoot masking protocol will provide greater interpretability of forefoot plantar pressure data, which will aid clinicians and researchers for diagnostic, prognostic, therapeutic and research purposes.

## Abbreviations

kPa: kilopascal
